# Evaluation of Osteoconduction of a Synthetic Hydroxyapatite/β-Tricalcium Phosphate Block Fixed in Rabbit Mandibles

**DOI:** 10.3390/ma13214902

**Published:** 2020-10-31

**Authors:** Luis Carlos de Almeida Pires, Rodrigo Capalbo da Silva, Pier Paolo Poli, Fernando Ruas Esgalha, Henrique Hadad, Letícia Pitol Palin, Ana Flávia Piquera Santos, Luara Teixiera Colombo, Laís Kawamata de Jesus, Ana Paula Farnezi Bassi, Carlo Maiorana, Roberta Okamoto, Paulo Sérgio Perri de Carvalho, Francisley Ávila Souza

**Affiliations:** 1 Implant Dentistry Post-Graduation Program, São Leopoldo Mandic School of Dentistry and Research Center, Campinas, SP 13 045 755, São Paulo, Brazil; luiscapires@hotmail.com (L.C.d.A.P.); fernandoesgalha@uol.com.br (F.R.E.); paulo.perri@unesp.br (P.S.P.d.C.); 2 Department of Diagnosis and Surgery, Araçatuba Dental School, São Paulo State University Júlio de Mesquita Filho—UNESP, Araçatuba, SP 16 015 050, São Paulo, Brazil; capalbo.rodrigo@gmail.com (R.C.d.S.); henriquehadad@gmail.com (H.H.); leticiappalin@gmail.com (L.P.P.); anaflaviaps_06@hotmail.com (A.F.P.S.); luara_colombo@hotmail.com (L.T.C.); kawamata_lais@hotmail.com (L.K.d.J.); ana.bassi@unesp.br (A.P.F.B.); francisley.avila@unesp.br (F.Á.S.); 3 Implant Center for Edentulism and Jawbone Atrophies, Maxillofacial Surgery and Odontostomatology Unit, Fondazione IRCSS Cà Granda Ospedale Maggiore Policlinico, University of Milan, 20122 Milan, Italy; carlo.maiorana@unimi.it; 4 Department of Basic Science, Araçatuba Dental School, São Paulo State University Júlio de Mesquita Filho—UNESP, Araçatuba, SP 16 015 050, São Paulo, Brazil; roberta.okamoto@unesp.br

**Keywords:** biocompatibility, biomaterials, bone augmentation, bone conduction, bone grafting, calcium hydroxyapatite, tissue regeneration

## Abstract

(1) Background: This study aimed to evaluate the incorporation of hydroxyapatite/β-tricalcium phosphate blocks grafted in rabbit mandibles. (2) Methods: Topographic characterization of biomaterial was performed through scanning electron microscopy coupled with energy-dispersive X-ray spectroscopy (SEM-EDX). Ten rabbits randomly received autogenous bone graft harvested from the tibia (Autogenous Group—AG) or synthetic biomaterial manufactured in β-tricalcium phosphate (Biomaterial Group—BG) at their right and left mandibular angles. Euthanasia was performed at 30 and 60 postoperative days; (3) Results: SEM-EDX showed a surface with the formation of crystals clusters. Histological analyses in BG at 30 days showed a slower process of incorporation than AG. At 60 days, BG showed remnants of biomaterial enveloped by bone tissue in the anabolic modeling phase. Histometric analysis showed that mean values of newly formed bone-like tissue in the AG (6.56%/9.70%) were statistically higher compared to BG (3.14%/6.43%) in both periods, respectively. Immunohistochemical analysis demonstrated early bone formation and maturation in the AG with more intense osteopontin and osteocalcin staining. (4) Conclusions: The biomaterial proved to be a possible bone substitute, being incorporated into the receiving bed; however, it showed delayed bone incorporation compared to autogenous bone.

## 1. Introduction

The results of alveolar bone remodeling after tooth loss, dentoalveolar trauma, and infections may promote an unfavorable site for dental implant rehabilitation with proper prosthetic planning [1]. Bone defects have different patterns and sizes, and their reconstruction can be classified into vertical and horizontal augmentation [2]. Several techniques have been proposed to treat bone defects, including bone substitutes from different origins such as autogenous, heterologous, and alloplastic materials [3,4].

In particular, block grafting is a recommended treatment in cases of horizontal defects, associated with severe atrophy of the alveolar bone [5,6,7,8]. In order to recreate the proper anatomy of the alveolar ridge, autogenous bone is considered the gold standard, being the only graft material that presents osteogenic, osteoinductive, and osteoconductive characteristics simultaneously [9]. On the other hand, the harvesting procedure is not free from drawbacks, including the risk of neurovascular injuries at the donor site, the need for an additional surgical site, an extended surgical time, an increasing post-operative patient morbidity, and a prolonged recovery time occasionally associated with hospitalization of the patient for extraoral donor sites [10]. Thus, research has been directed towards the use of biomaterials as autogenous bone substitutes when reconstructive surgery is needed. Currently, a wide range of synthetic or biological biomaterials is commercially available. 

Among the different biomaterials, beta-tricalcium phosphate (β-TCP) ceramic has been introduced as a bone substitute [11]. β-TCP acting as a scaffold promotes bone formation through osteoconduction. As a result of its biocompatibility, bioactivity, favorable incorporation, and osteoconductive properties, synthetic β-TCP has been generally accepted and evaluated as a grafting material [12,13,14]. Its structural characteristic resembling the mineral phase of bone tissue is another factor that promotes great expectations for this material as a bone substitute [15]. As a disadvantage, biomaterials based on β-TCP present high solubility, which makes them unsuitable in reconstructions that need to maintain and create bone volume [16]. In order to reduce its rapid absorption, the association of β-TCP with hydroxyapatite (HA) in different proportions has been studied [17]. It was found that the biphasic composition consisting of HA and β-TCP provides better osteoconductive capabilities when compared to monophasic ceramics, decreasing osteoclastic activity allowing the formation of a framework for osteoconduction [18]. The search for the ideal ratio of HA and β-TCP, although controversial, is considered important because it modifies the solubility of the biomaterial and influences the resorption time. In a biocompatibility and biomechanical study, the association of 60%/40% HA/β-TCP has been tested and suggested as the ideal proportion of these components [19]. The combination of 60% HA/40% β-TCP has been used in a study to lift maxillary sinus in humans, obtaining favorable results for bone neoformation, proving to be an interesting ratio [20]. Different experimental studies in animals and clinical trials have demonstrated the favorable incorporation of such biphasic composition in the filling of critical defects in the calvarium, maxillary sinus, and alveolar socket preservation after dental extraction [14,21,22]. However, most studies employing β-TCP were performed in the reconstruction of two or more walls filled with cements or granular biomaterials, which may promote bone neoformation by taking advantage of the osteoconductive potential of the material. On the contrary, few studies are currently available that evaluate the incorporation of this biomaterial in the form of blocks for horizontal alveolar ridge augmentation [23,24].

In view of the above, the purpose of the present study was to analyze and compare the process of bone incorporation of autogenous bone graft and 60% HA/40% β-TCP blocks fixed by means of bicortical screws to the mandibular angles of rabbits.

## 2. Materials and Methods

### 2.1. Study Design

The study protocol was approved by the local Animal Experimentation Ethical Committee (CEEA) with the identification number 00999/2011. The sample size calculation was based on histometric and immunohistochemistry values retrieved from the authors’ pilot analyses (unpublished data) before the commencement of the study. New bone formation was determined as the primary outcome. Thus, a significant difference of 5% (standard deviation of 2%) was considered, and for 80% power and setting alpha at 0.05, five grafts per group were necessary in order to compare two different groups. 

The sample consisted of 10 male New Zealand white adult rabbits with a weight ranging from 3 to 4 kg. The animals were kept in individual cages in a humidity- and temperature-controlled environment, on a light-dark cycle (12:12 h), and fed with water and rabbit chow (Procoelho-Primor, São Paulo, SP, Brazil) ad libitum. The surgical procedures were made at the local Rabbit Experimentation Center. After randomization using computer-generated lists on the website Research Randomizer (https://www.randomizer.org/), the animals received the bone blocks in their mandibles according to a split-mouth model as described below. 

In the autogenous group (AG), an autogenous bone block harvested from the medial portion of the left tibial metaphysis was randomly grafted into the left or right mandibular angle. In the biomaterial group (BG), the synthetic biphasic calcium phosphate biomaterial (Grafts^®^ BCP, Smart Bone Substitute, Aix-En-Provence, France) in the form of blocks was randomly grafted into the contralateral mandibular angle. The biomaterial was composed of a stable component (HA) and a bioactive bioabsorbable component (β-TCP) in a 60:40 ratio, respectively. The rationale was to provide a good balance between support for cell growth and graft absorption. The pores greater than 100 µm distributed over the entire surface of the material provided a degree of porosity of 70%. The relationship between porosity and the HA/TCP ratio represented a novelty that could provide greater cellular activity and maintenance of the trabecular architecture of the biomaterial. 

### 2.2. Topographic Characterization of the Biomaterial

After covering the biomaterial with a thin layer of carbon, the surface topography of the biomaterial was analyzed using an electron microscope (SEM ZEISS, model EVO LS15, Oberkochen, Germany) to analyze the biomaterial surface morphology and its porosity, coupled with the X-ray dispersive energy spectroscopy system (Oxford model EDX microanalysis detector, Inca X-act, Oxford instruments, Abingdon, UK), for semi-quantitative analysis of the chemical composition of surfaces.

### 2.3. Experimental Surgery

**Figure 1:**
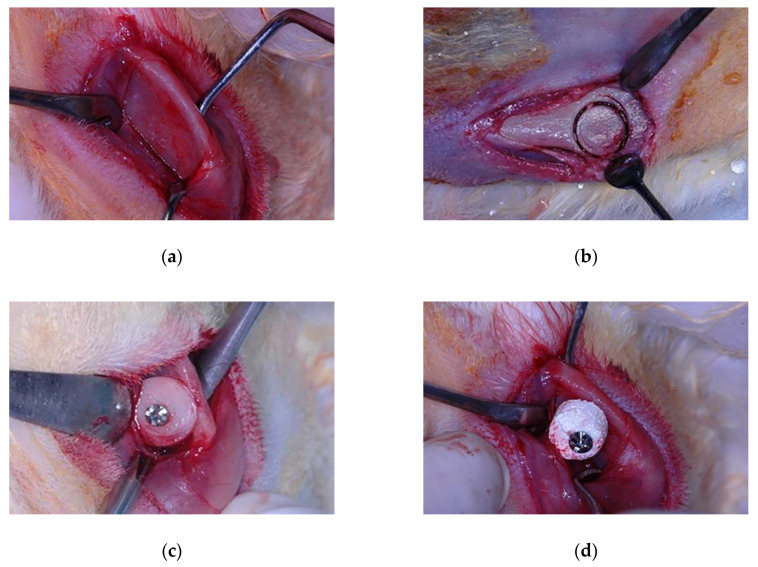
Experimental surgery procedure: (**a**) Surgical exposure of the recipient bed; (**b**) Autogenous bone block grafting procedure; (**c**) Autogenous block grafted in the autogenous group; (**d**) Biomaterial block grafted in the biomaterial group.

General anesthesia was induced by intramuscular injections of ketamine hydrochloride (Vetaset—Fort Dodge Saúde Animal Ltd., Campinas, São Paulo, Brazil) at a dose of 50 mg/kg body weight associated with xylazine hydrochloride (Dopaser—Laboratório Calier do Brasil Ltd., Osasco, São Paulo, Brazil) at a dose of 5 mg/kg body weight. Subsequently, trichotomy and topical antisepsis were performed in both mandibular angle regions and in the left tibia with iodine solution (PVPI 10%, Riodeine, Rioquímica, São José do Rio Preto, Brazil). In addition, local infiltration of 0.3 mL/kg of mepivacaine hydrochloride (Scandicaine 2% with epinephrine 1:100,000, Septodont, Saint Maur des Fossés, France) was used as local anesthesia. A 2-cm dermal incision was made with a #15 scalpel blade (Feather Industries Ltd., Tokyo, Japan) mounted on a #3 scalpel handle in correspondence with each mandibular angle. Soft tissues were dissected respecting tissue planes reaching the periosteum. The periosteal tissue was finally incised in order to expose the bone tissue of the recipient area (Figure 1a). Bone decortication of the lateral plate of the mandibular angle was performed with a #701 rotary drill (Maillefer Instruments, Ballaigues, Switzerland), mounted on a straight surgical handpiece (Kavo do Brasil, Joinvile, Brazil), under constant irrigation of 0.9% sterile saline solution (Darrow, Rio de Janeiro, Brazil). In the AG, following the exposure of the recipient area, autogenous bone was harvested from the tibia. A 2-cm linear incision was made in correspondence with the left tibial metaphysis. A full-thickness skin flap, including skin, muscle, and periosteum, was raised exposing the surface of the bone. A circular osteotomy was performed (Figure 1b) by means of 8-mm internal diameter trephine bur (Neodent^®^, Curitiba, Paraná, Brazil) mounted on a contra-angle handpiece with a 20:1 reduction (Kavo^®^ do Brasil, Joinvile, Brazil) at a speed of 1500 rpm under constant irrigation with 0.9% sodium chloride (Darrow, Rio de Janeiro, Brazil). The bone was carefully removed from the trephine and preserved in a sterile saline solution. In the BG, biphasic calcium phosphate ceramic blocks were harvested using the same trephine size, in order to collect grafts of the same diameter in both experimental groups. Autogenous (Figure 1c) or synthetic block grafts (Figure 1d) were subsequently perforated with a 1.2 mm-diameter drill under copious irrigation with sterile saline approximately in their central portion, and randomly fixed to the left or right mandibular angle by means of compressive 1.6 × 8 mm osteosynthesis bicortical screws (SIN, Sistema de Implante Nacional, São Paulo, Brazil) resulting in appositional onlay-like block graft. The recipient bed was perforated with a 1.0 mm-diameter drill cooled with sterile saline in order to favor nourishment and revascularization of the block graft.

Soft tissues were carefully repositioned and sutured in different layers for primary wound closure using an absorbable suture (Polyglactin 910—Vicryl 4.0, Ethicon, Johnson Prod., São José dos Campos, SP, Brazil), while a non-resorbable monofilament suture (Nylon 4.0, Ethicon, Johnson, São José dos Campos, SP, Brazil) was used for interrupted skin suturing. Additional antisepsis with PVPI was conducted after the suturing. Intramuscular pentabiotic (0.1 mL/kg, Fort Dodge Saúde Animal Ltd., SP, Brazil) was injected immediately after the surgery. A single dose of sodic dipyrone (1 mg/kg/day, Ariston Indústrias Químicas e Farmacêuticas Ltd., São Paulo, SP, Brazil) was also administered. Neither food nor movement restriction was applied to the animals that remained in individual cages during the experimental period. At 30 and 60 postoperative days, 5 animals in each group, respectively, were sacrificed by a lethal dose of pentobarbital (200 mg/kg). The soft tissues were then dissected, and the mandible of each rabbit was extracted. Osteotomies were performed on each mandible to obtain bone samples with at least 3 cm of margins circumferentially around the grafted area. All samples were immersion-fixed in 10% neutral buffered formalin (Reagentes Analíticos^®^, Dinâmica Odonto-Hospitalar Ltd., Catanduva, SP, Brazil).

### 2.4. Histological Laboratorial Processing

After fixing and washing for 24 h in running water, the descaled were decalcified in 20% EDTA (ethylenediaminetetraacetic acid, Merck, Darmstadt, Germany) dissolved in Milli-Q water, replaced weekly for a period of 6 weeks, at room temperature. Following decalcification, the osteosynthesis screws were carefully removed. The samples were then dehydrated in an ascending series of alcohol concentrations (70, 90, 95, and absolute alcohol), changing the solutions every hour in an orbital shaker (KLine CT—150^®^, Cientec—Equipamentos para Laboratório, Piracicaba, SP, Brazil). The samples were successively cleared in xylol, paraffin-embedded, and prepared using a precision saw to obtain 5 μm thick sections. The sections were mounted onto slides and stained with hematoxylin eosin (HE Merck & Co., Inc., Kenilworth, NJ, USA) for qualitative and histometric analysis, and stained by osteopontin (OP), osteocalcin (OC), and Tartrate-resistant Acid Phosphatase (TRAP) for the immunohistochemical analysis.

### 2.5. Qualitative Histological Analysis

Five slides of each sample were obtained for hematoxylin and eosin staining in the most central region of the piece. The qualitative evaluation was performed by a researcher, blinded with respect to the experimentation through a binocular optical microscope JENAMED 2 (Carl-Zeiss, Oberkochen, Germany), considering the internal part of the bone graft, the interface between the bone graft and the recipient bed, and the newly formed tissue.

### 2.6. Histometric Analysis

The capture of the region of interest involving the bone substitute/autogenous bone block, the interface between the graft and the recipient bed, and the recipient bed, was performed with a 40× magnification, considering the most central region identified by the contour of the fixation screw and the limits of the graft. The capture generated an image of the bone substitute, the recipient bed, and their interface. After obtaining the images, the quantitative analysis was performed using dedicated software (Image J version 1.53, NIH Image, Bethesda, MD, USA). The image and respective percentage value generated by the capture using the “rectangle” tool was considered as the total area to be evaluated. After that, through the integrated tool “polygon”, the bone substitute, connective tissue, and newly formed bone tissue areas were delimited and the respective percentage areas proportional to the total area were calculated and submitted to statistical tests.

### 2.7. Immunohistochemical Analysis

The immunohistochemical analysis was performed in the Department of Basic Sciences, Araçatuba Dental School—UNESP (FAPESP, 2015/14688-0). The histological slices obtained after 30 and 60 days from the surgical procedure were used.

The immunohistochemical procedure started by deparaffinization and rehydration of the slices, and then endogenous peroxidase activity was inhibited with hydrogen peroxide. Endogenous biotin was blocked with skimmed milk. The primary antibodies (Santa Cruz Biotechnology, Inc., 10410 Finnell Street, Dallas, TX 75220 USA) were used against Osteopontin (OP—SC10593), Osteocalcin (OC—SC18319), and Tartrate-resistant Acid Phosphatase (TRAP—SC30832). OP and OC proteins are expressed during the mineralization process, where OP marks osteoblasts at the beginning of the mineralization process and OC is expressed in the late stages of the mineralization process. TRAP, on the other hand, is expressed by osteoclast activity, allowing the evaluation of the resorption process. The rabbit anti-goat IgG (H+L) secondary antibody, Biotin (Pierce Biotechnology, Waltham, MA, USA) was used and the reaction signal was amplified by streptavidin (Dako North America, Inc., 6392 Via Real Carpinteria, CA 93013, USA). The Diaminobenzidine (Dako North America, Inc., 6392 Via Real Carpinteria, CA 93013, USA) ends the reaction and then the slices were counterstained with Meyer Hematoxylin.

For each antibody used, the expression of proteins was evaluated semi-quantitatively by assigning different “scores” in accordance with the immunostained cells in the wound-healing process. The analysis was performed with an optical microscope (LeicaR DMLB, Heerbrugg, Switzerland) by means of scores (ordinal qualitative analysis); when the scores were light labeling (++), moderate labeling (+++), and intense labeling (++++) it was considered positive for diaminobenzidine, taking care to hold negative controls to evaluate the specificity of the antibodies. These scores and the methodology were established according to previous studies [25,26,27], where light labeling represented about 25% of the immunolabeling area in the blades, moderate labeling represented about 50%, and intense labeling represented about 75%.

### 2.8. Statistical Analysis

An independent statistician performed the statistical analysis using IBM SPSS Statistics 24.0 (IBM Corp., Armonk, NY, USA). The results were initially submitted to the Shapiro–Wilk test to assess data distribution. After testing for normality, Student’s *t*-test was performed for parametric data, while the Mann–Whitney U test was applied in cases of non-parametric data. A significance level of 0.05 was adopted for all tests.

## 3. Results

### 3.1. Topographic Characterization of Biomaterial

**Figure 2:**
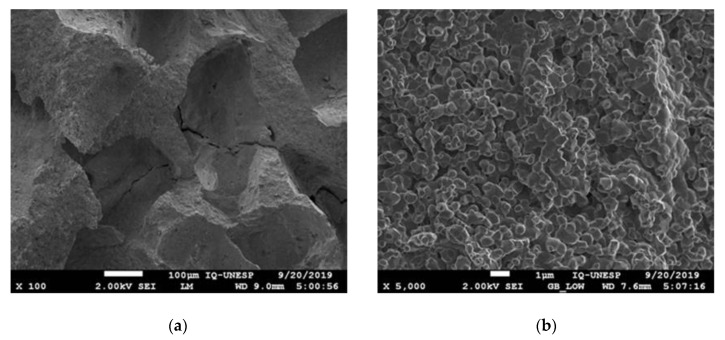

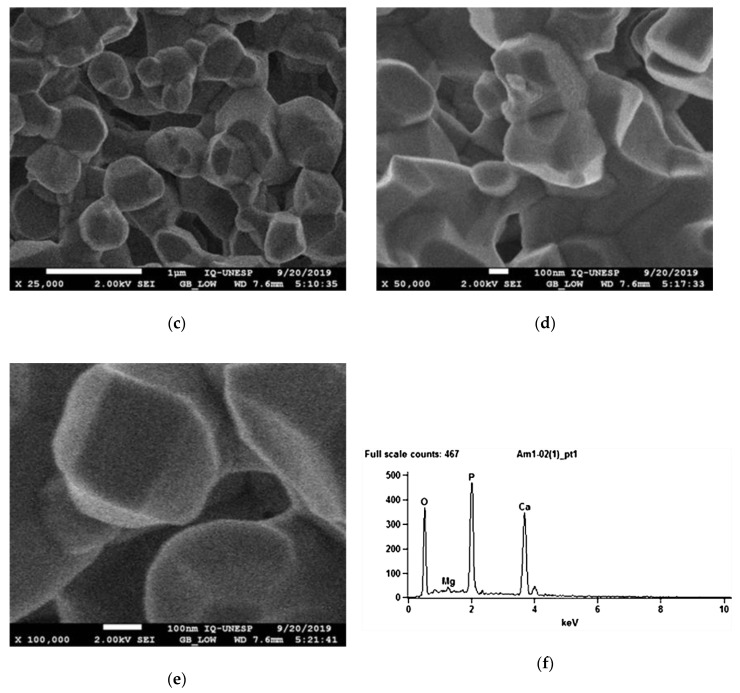
SEM-EDX analysis: (**a**–**e**) SEM views of the biomaterial surface at ×100, ×5000, ×25,000, ×50,000, and ×100,000 magnifications, respectively. (**f**) EDX spectrometry values obtained before the surgery. The elements found were phosphorus, oxygen, calcium, and magnesium.

The SEM-EDX analysis evidenced a surface with the formation of crystals clusters and high macroporosity > 100 μm between it. The pores showed a crater shape and approximately 100 µm to 200 µm in diameter in multidirectional ways (Figure 2a). It was possible to observe regions with different heights on the same block surface (Figure 2b). The biomaterial base showed irregularities of the surface accompanied by its porosity, as well as the crystal clusters in different shapes and sizes (Figure 2c–e). The X-ray Dispersive Energy Spectroscopy analysis showed the amount of phosphorus (467), oxygen (372), and calcium (365) ions present, respectively, followed by a low percentage of magnesium (Figure 2f).

### 3.2. Qualitative Histological Analysis

- Autogenous group

**Figure 3:**
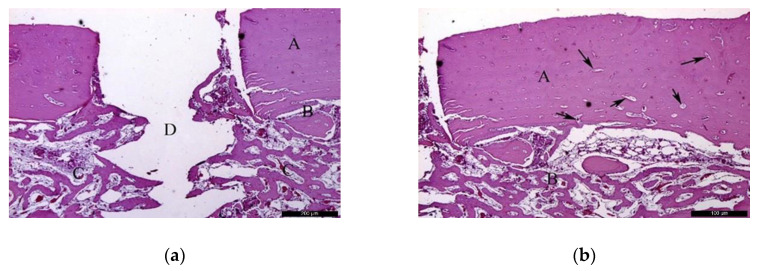
Histological analysis of the autogenous group (AG) at 30 days. (**a**) Autogenous bone graft (A) positioned over the recipient bed (B), trabeculae in maturation stage and good vascularization (C); space previously occupied by the osteosynthesis screw is recognizable in (D). Hematoxylin and eosin stain at a magnification 40×; (**b**) Autogenous bone (A) newly formed bone (B), and small areas of resorption (black arrows). Hematoxylin and eosin stain at a magnification 125×.

In the AG, after 30 healing days (Figure 3a), it was possible to observe the autogenous bone graft (A), and bone trabeculae under the receiving bed (B). The space between the graft and the recipient bed was occupied by bone tissue with a relevant number of trabeculae in the maturation stage with high cellular activity and good vascularization (C). Focusing on the graft (Figure 3b), autogenous bone (A) and newly formed bone (B) could be identified. In the inner part of the autogenous bone graft, small areas of resorption (black arrows) might indicate a phase of remodeling.

**Figure 4:**
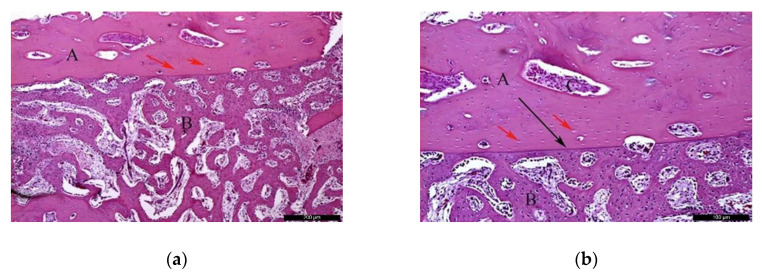
Histological analysis of the AG at 60 days. (**a**) Autogenous bone graft (A) in contact with the newly formed bone (B), and anucleated cells suggesting the presence of osteoblasts (red arrows). Hematoxylin and eosin stain at a magnification 40×; (**b**) Autogenous bone graft (A) and newly formed bone (B) separated by a cement line (black arrow) and osteocytes (red arrows). Hematoxylin and eosin stain at a magnification 125×.

In the AG, after 60 post-operative days (Figure 4a), it was possible to observe the autogenous bone graft (A) in contact with the newly formed bone (B). Internally, the presence of connective tissue areas might suggest a remodeling phase of the bone. The identification of anucleated cells could suggest the presence of osteoblasts (red arrows). At the microscopic examination (Figure 4b), it was possible to distinguish the autogenous bone graft (A) and newly formed bone (B) separated by a cement line (black arrow). In the inner part of the autogenous bone graft, connective tissue areas were noticed, confirming the remodeling phase of the bone. Osteocytes were clearly visible (red arrows).

- Biomaterial group

**Figure 5:**
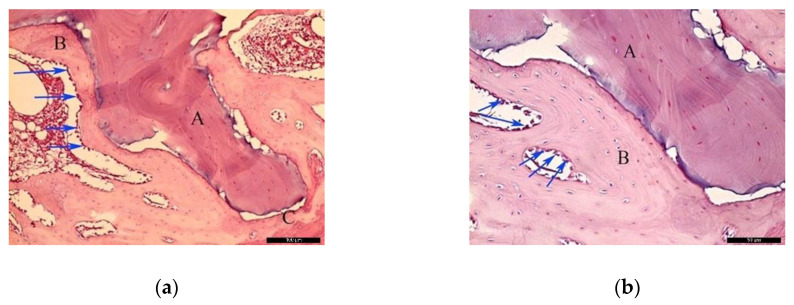
Histological analysis of the biomaterial group (BG) at 30 days. (**a**) Biomaterial (A) in close contact with the newly formed bone (B), and the residual recipient site (C); osteoblast-like lining cells are indicated with blue arrows. Hematoxylin and eosin stain at a magnification 40×; (**b**) Biomaterial (A), newly formed bone (B), and lining osteoblast cells in the periphery of the newly formed bone (blue arrows). Hematoxylin and eosin stain at a magnification 125×.

In the BG, after 30 healing days (Figure 5a), it was possible to observe the biomaterial (A) in close contact with the newly formed bone (B), and the residual recipient site (C). In the surrounding areas of the neoformed bone, it was possible to identify a high number of lining cells compatible with osteoblasts (blue arrows) suggesting a phase of matrix synthesis. At higher magnification (Figure 5b), the contiguous contact between the biomaterial (A) and the neoformed bone (B) was appreciated. Lining osteoblast cells were identified in the periphery of the newly formed bone (blue arrows).

**Figure 6:**
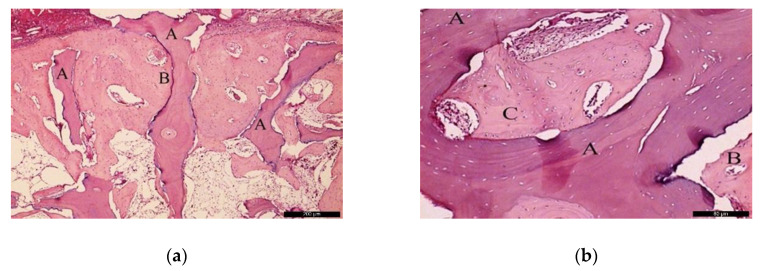
Histological analysis of the BG at 60 days. (**a**) Biomaterial (A) enwrapped by newly formed bone (B). Hematoxylin and eosin stain at a magnification 40×; (**b**) Biomaterial (A) on the recipient bed (B), and newly formed bone (C). Hematoxylin and eosin stain at a magnification 125×.

In the BG, after 60 post-operative days (Figure 6a), the biomaterial (A) was enwrapped by neoformed bone (B). In the inner part of the biomaterial, it was possible to observe islands of newly formed bone, highlighting the osteoconductivity capability of the bone substitute. At the same time, areas of resorption associated with volume loss were noticed, indicating high solubility of the biomaterial. At higher magnification (Figure 6b), it was possible to observe the bone substitute (A) on the recipient bed (B), and neoformed bone tissue (C).

### 3.3. Histometric Analysis

The mean values of the neoformed bone tissue in the AG were 6.56% and 9.70% at 30 and 60 postoperative days, respectively, while the mean values of connective tissue were 47.83% and 45.34% in the same periods evaluated. Additionally, the mean values of autogenous graft remnants were 45.61% and 44.96%, respectively. For the BG, the mean values of newly formed bone tissue were 3.14% and 6.43% at 30 and 60 postoperative days, respectively, while the mean values of connective tissue were 41.58% and 45.34% in the same periods analyzed. The mean remaining values of the synthetic biomaterial based on β-tricalcium phosphate were 55.28% and 40.26%, respectively. The registered data were submitted to statistical analysis, namely *t*-test for parametric data (neoformed bone tissue, and bone substitute in both periods of analysis, and connective tissue at 60 days period), and Mann–Whitney test for non-parametric data (connective tissue at 30 days period). The mean values of neoformed bone tissue in the AG were statistically higher when compared to the mean values of neoformed bone tissue in the BG at both experimental periods. Conversely, there was no statistically significant difference between groups in the amount of connective tissue and bone substitute in both experimental periods. The data of newly formed bone tissue, connective tissue, and remnants of autogenous graft and biomaterial for both groups are reported in Table 1.

### 3.4. Immunohistochemical Analysis

At 30 postoperative days, in both groups, it was possible to observe bone tissue in the ongoing maturation phase, with neoformed bone trabeculae, and the presence of osteoblastic lineage cells. Already at 60 days, in both groups, organized, mature bone tissue, with the presence of osteocytes inside the gaps surrounded by a mineralized bone matrix, was noticed.

**Figure 7:**
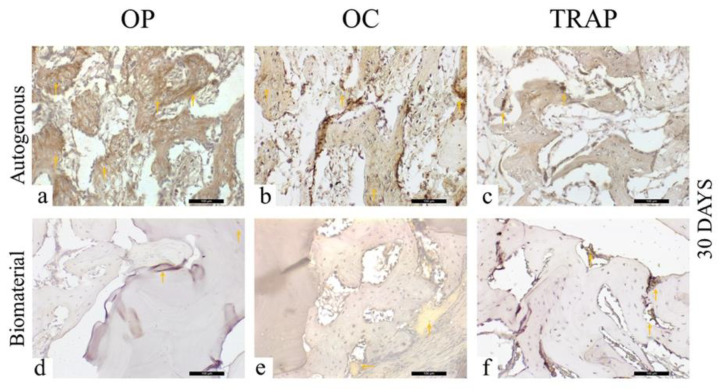
Osteopontin (OP), Osteocalcin (OC), and Tartrate-resistant Acid Phosphatase (TRAP) immunostaining at 30 days for the experimental groups. Immunolabeling is indicated by orange arrows. (**a**) OP immunostaining at 30 days in the AG; (**b**) OC immunostaining at 30 days in the AG; (**c**) TRAP immunostaining at 30 days in the AG; (**d**) OP immunostaining at 30 days in the BG; (**e**) OC immunostaining at 30 days in the BG; (**f**) TRAP immunostaining at 30 days in the BG.

In the AG, at 30 days, it was possible to observe an intense osteopontin staining, showing a very intense (++++) initial bone mineralization activity, with positivity for the cells (osteoblasts) around the trabeculated bone, as well as the staining itself in the mineralized extracellular matrix (Figure 7a). In contrast, in the same period, in the BG, a light marking (++) was observed (Figure 7d), with a scarce mineralized extracellular matrix.

Regarding osteocalcin, a protein related to the late stage of mineralization of the extracellular matrix, a moderate (+++) staining was observed in the AG (Figure 7b), with cells of the osteoblastic lineage and the extracellular matrix presenting positive immunostaining for this protein. When the osteopontin and osteocalcin marking was correlated for this experimental group, it was observed that the bone mineralization activity was in the initial stages, compatible with the evaluation period (30 days). In the BG, it was possible to notice positive osteocalcin light (++) staining in the pre-existing bone tissue, with osteoblast lineage cells and a positively stained extracellular matrix (Figure 7e).

The TRAP expression, showing osteoclasts in bone resorption activity, was light (++) in the AG (Figure 7c), with low osteoclast activity, and was moderate (+++) for the BG (Figure 7f), indicating resorption activity.

**Figure 8:**
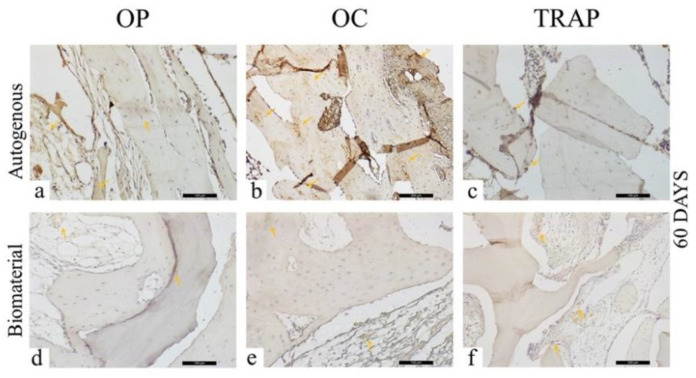
Osteopontin (OP), Osteocalcin (OC), and Tartrate-resistant Acid Phosphatase (TRAP) immunostaining at 60 days for the experimental groups. Immunolabeling is indicated by orange arrows. (**a**) OP immunostaining at 60 days in the AG; (**b**) OC immunostaining at 60 days in the AG; (**c**) TRAP immunostaining at 60 days in the AG; (**d**) OP immunostaining at 60 days in the BG; (**e**) OC immunostaining at 60 days in the BG; (**f**) TRAP immunostaining at 60 days in the BG.

At 60 days, in the AG, a moderate (+++) osteopontin staining was observed (Figure 8a), with the presence of positively stained cells adjacent to the bone tissue. It is worth noting the organization of the bone tissue visible in the region of interest. At the same time, osteocalcin was markedly intense (++++), with areas of precipitation on mature bone tissue (Figure 8b). Positively marked osteocytes were noted, characterizing the maturity of the bone tissue in this period. TRAP protein was lightly (++) marked, with few osteoclasts in resorption activity (Figure 8c).

For the BG, light (++) staining was observed for osteopontin, osteocalcin, and TRAP, showing few cells stained for each of these proteins (Figure 8d–f). Regarding osteopontin and osteocalcin, which are extracellular matrix proteins presenting staining on the mineralized matrix, the low mineralization activity for both proteins in this later period stands out. There were also few TRAP stained in the region of interest.

The scores obtained from the immunohistochemical analysis in the periods of 30 and 60 days are listed in Table 2 and Table 3, respectively.

## 4. Discussion

The ability of bioceramics to interact with osteoblastic cells is essentially related to their structural properties. Synthetic β-calcium-phosphate-based biomaterials present a porosity of approximately 70%. This type of conformation provides a considerable surface area supplied with several protein-binding sites available for a stable adhesion with a high number of osteoblasts [28]. Another factor that must be considered is the pore diameter. A previous study showed that wide pores with an accessible volume (>200 μm) exhibited an incremented proliferation and differentiation of osteoblasts within the scaffold, as a result of the revascularization process supplying oxygen and nourishment [29]. The same concept can be applied to calcium-phosphate-based biomaterials, in which the presence of pores allows the migration and proliferation of osteoblasts and mesenchymal cells along with the revascularization process needed to promote bone formation. In addition, a porous surface enhances the interconnection between the biomaterial and the surrounding bone tissue, creating high mechanical stability in the recipient bone–biomaterial interface [30]. The SEM showed a macroporosity > 100 μm of the synthetic biomaterial employed in the present study, which constitutes approximately 45% of its composition, thus facilitating the biological exchanges needed for the osteogenic process. Conversely, such porosity led to technical difficulties during the stabilization of the graft to the recipient bed during the experimental surgery. Higher strength was needed to tighten the screws while fixing the graft, with the risk of developing cracks and fractures of the blocks. Consequently, special attention was paid while fixing the biomaterial to the recipient bone in order to prevent block fractures during the intra- and post-operative periods. Indeed, the drilling technique in cases of such small-sized blocks is extremely challenging, especially for the biomaterial group, as the bone substitute is very friable. In addition, from an anatomical standpoint, the mandibular angle is particularly fragile, and requires care to obtain an effective fixation. Thus, the perforations were made in a direction that guaranteed the preservation of the block and the recipient bed in order to avoid complications such as the fracture of the block or the loss of stability in the fixation. This, however, often resulted in a non-centered position of the osteosynthesis screw, which may be considered a technical limitation during the histomorphometric analysis, as a central position of the screw would have been more helpful by serving as a reference guide during the cutting process. Energy-dispersive X-ray spectroscopy showed the presence of a low percentage of magnesium on the biomaterial surface. This component can promote the osteoimmunomodulatory properties, by improving osteoinduction by BMP signaling [31], being considered essential for bone tissue [32,33].

Autogenous bone graft is characterized by a relevant number of advantages with respect to most of the bone substitutes, particularly in terms of biocompatibility and regenerating potential. Autogenous bone is considered the gold standard due to its osteogenic properties, in addition to osteoinductive and osteoconductive capabilities, promoting the apposition of new bone tissue from the pre-existing bone as a model for new bone formation. In the present study, such properties have been validated when analyzing the microscopic images of the autogenous block graft at 60 post-operative days. Close contact between the recipient bed and the grafted autogenous block was observed, together with the presence of cement lines, newly formed bone associated with a high number of trabeculae, and vital osteocytes populating the inner portions of the graft. These biological events were promoted by the bone matrix, which induced the differentiation of osteoprogenitor cells of the recipient region into osteoblasts in order to stimulate bone formation. Those advantages and characteristics are supported by the expected results of the histometric analysis, where the AG presented a statistically significant difference in bone neoformation when compared to the BG. However, the autogenous graft needs a donor site, which contributed to the development of biomaterials, such as the HA-β-TCP used in the present experimental study, and although the histometric findings showed a greater amount of newly formed bone (bone incorporation) for the AG, the biomaterial was incorporated into the recipient bed and allowed bone formation by osteoconduction, which was expected. A previous study compared autogenous bone grafts with allogeneic grafts in block form through histological, histometric, and immunohistochemistry analyzes, showing the superiority of the autogenous bone in new bone formation and maturation of bone tissue during the incorporation process [34]. This highlights the need to search for an ideal biomaterial as an alternative to autogenous grafts.

Histological findings were confirmed by the immunostaining of OP and OC proteins. The marking of OP was intense at 30 days in the AG and light in the BG, showing earlier osteoblastic activity in the former. A delayed incorporation is confirmed within 60 days, when AG showed intense osteocalcin expression indicating bone maturation, whereas BG showed light expression of this protein, which represents the formation of an immature tissue in this period. In a previous study assessing critical-size defects in rat calvaria filled with autogenous bone or beta-tricalcium phosphate biomaterial, the authors evaluated the immunostaining of osteocalcin at 30 and 60 postoperative days [13]. It was observed that the AG showed higher protein expression at 60 days, indicating a greater maturation of the bone tissue formed compared to the biomaterial, which is in agreement with the results obtained herein.

Considering the donor site, the rabbit tibia was used to collect the autogenous graft. The reason is related to low post-operative morbidity, and ease of the surgical technique with respect to other donor sites. Harvesting bone from the rabbit calvaria leaves the dura-mater exposed due to an inability to separate the external and the internal cortical plates. The tibia is mainly characterized by cortical bone, with the presence of an intra-medullary canal. Hence, due to its composition, the tibia could be safely used to retrieve bone tissue in the form of blocks, with an adequate thickness for bone reconstructions [35]. On the other hand, in order to reduce the morbidity associated with the harvesting procedure, the research has been directed toward the use of bone substitutes. Among the wide range of biomaterials, synthetic bioceramics are gaining space in clinical scenarios as an alternative to autogenous bone grafts in maxillo-facial reconstructions. This study evaluated the incorporation of a biomaterial based on beta-tricalcium phosphate with hydroxyapatite compared to autogenous bone grafted in rabbit mandibles, in order to simulate critical clinical situations requiring horizontal augmentation in mandibular alveolar ridges, in which the use of biomaterial blocks may be indicated. However, for vertical defects, as an alternative to autogenous bone grafts, these biomaterials may not be the best clinical choice, or even contraindicated.

The biocompatibility and bifunctionality of calcium phosphate bioceramics in the substitution of bone tissue have been evaluated in vivo [36]. It was concluded that HA and β-TCP were progressively resorbed and substituted with newly formed bone, acting as a scaffold that could be favorably colonized by osteogenic cells. β-TCP has been compared to biphasic calcium phosphate ceramic containing HA/β-TCP in a 75:25 ratio in the process of trabecular bone repair in an animal model [30]. The histomorphometric analysis demonstrated that 36% of β-TCP, used alone, underwent resorption in 26 weeks, whereas the HA/β-TCP group healed undisturbed. Bone neoformation occurred in both groups; however, a higher amount of peripheral bone volume was observed in the biphasic ceramic group. Therefore, it was concluded that both bone substitutes were biocompatible and were characterized by a satisfying osteoconductive potential; nevertheless, the biodegradation of β-TCP was faster, losing the volume that should be maintained. A previous study evaluated β-TCP as a bone substitute in preserving the alveolar ridge for subsequent implant insertion in humans [37]. The ridge dimensions were measured before the grafting procedure and after a 6-month healing period at the second stage surgery for tissue sample harvesting and implant placement. Resorption of approximately 9% occurred during the healing time. After 6 months, the histological evaluation showed a complete resorption of the biomaterial, replaced by vital bone of good quality and quantity, which allowed a proper implant insertion. This might underline the osteoconductive properties of the biomaterial associated with a high level of solubility. Accordingly, the same pattern has been observed during the histological analysis of the present study. However, it must be noticed that the biomaterial has been used in a more challenging clinical situation in the present study, namely, as an appositional block graft to increase the bone thickness, and not as a filling material in self-containing defects during socket preservation procedures. In addition, a recent study evaluated HA/β-TCP in the form of blocks [38]. The study assessed and compared qualitatively autogenous bone grafts versus a synthetic biomaterial based on HA/β-TCP in a 60:40 proportion. The biomaterial underwent gradual resorption followed by the apposition of bone tissue. Nonetheless, even at 60 post-operative days, it was possible to detect a certain amount of bone substitute in contact with newly formed bone and the recipient bed.

## 5. Conclusions

In conclusion, the biomaterial based on β-tricalcium phosphate combined with hydroxyapatite evaluated in the present experimental study served as a scaffold, promoting bone formation in the grafted area and highlighting its osteoconductive property. However, there was a delay in the incorporation process when compared to the autogenous block graft.

## Figures and Tables

**Table 1:** Analysis of the comparison between the biomaterial and autogenous bone at each experimental period.

Period	Group	Bone Substitute	Connective Tissue	Newly Formed Bone
30 days	Biomaterial Autogenous	55.28% 4.61%	41.58% 47.83%	3.14% * 6.56% *
60 days	Biomaterial Autogenous	40.26% 44.96%	53.31% 45.34%	6.43% * 9.70% *

* Statistically significant difference between BG and AG.

**Table 2:** Osteopontin (OP), Osteocalcin (OC), and Tartrate-resistant Acid Phosphatase (TRAP) immunostaining at 30 days for the experimental groups.

30 Days	Autogenous Group	Biomaterial Group
OP	++++	++
OC	+++	++
TRAP	++	+++

**Table 3:** Osteopontin (OP), Osteocalcin (OC), and Tartrate-resistant Acid Phosphatase (TRAP) immunostaining at 60 days for the experimental groups.

30 Days	Autogenous Group	Biomaterial Group
OP	+++	++
OC	++++	++
TRAP	++	++
